# Supplementary material for: BRCA2 chaperones RAD51 to single molecules of RPA-coated ssDNA
Source: Proc Natl Acad Sci U S A. 2023 Mar 28;120(14):e2221971120. doi: 10.1073/pnas.2221971120 (PMC10083600; doi:10.1073/pnas.2221971120)
Supplement: Supplementary file 1 — Appendix 01 (PDF) [file pnas.2221971120.sapp.pdf]

PNAS

Supporting Information for

BRCA2 chaperones RAD51 to single molecules of RPA-coated ssDNA

Jason C. Bell[1,2*], Christopher C. Dombrowski[1,2*], Jody L. Plank[1,2], Ryan B. Jensen[1,2,3], and Stephen C. Kowalczykowski[1,2,†]

[1]Department of Microbiology & Molecular Genetics and [2]Department of Molecular & Cellular Biology, University of California, Davis, California 95616, USA, [3]Department of Therapeutic Radiology, Yale University School of Medicine, New Haven, Connecticut 06520, USA

*These authors contributed equally to this work.

[†]Correspondence: S.C.K.: sckowalczykowski@ucdavis.edu, Tel. (530) 752-5938

This PDF file includes:

Figures S1 to S2
Legends for Movies S1 to S3

Other supporting materials for this manuscript include the following:

Movies S1 to S3

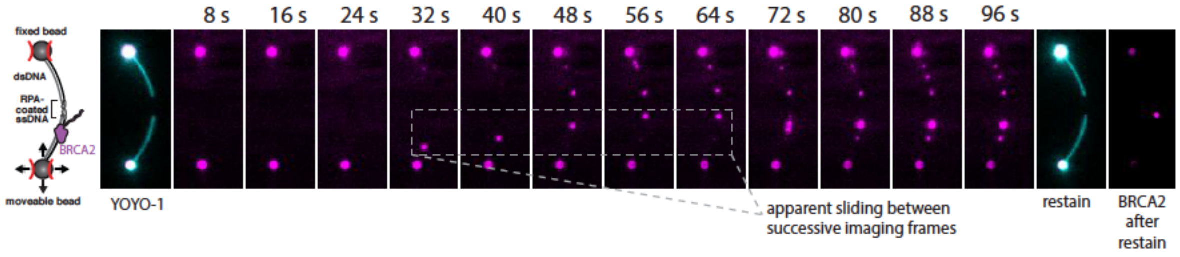

Supplementary Figure 1: Movement of BRCA2 under flow observed on dsDNA. Montage of a single gapped λDNA molecule held between two optically trapped polystyrene beads in a 6-channel flow cell as described in Figure 1. The molecule was imaged in Channel 5 and iteratively dipped into Channel 6, containing BRCA2 (purple, α-MBP^{AF546}), by moving the molecule (*down, then up*) perpendicular to the flow (from *left to right*) between images in the montage. The BRCA2 focus highlighted between 32 s and 64 s slides upwards towards the junction between the dsDNA and RPA-coated ssDNA. At the end of the movie, the gapped λDNA was re-stained in Channel 3, whereupon the less stable BRCA2-dsDNA complexes dissociated.

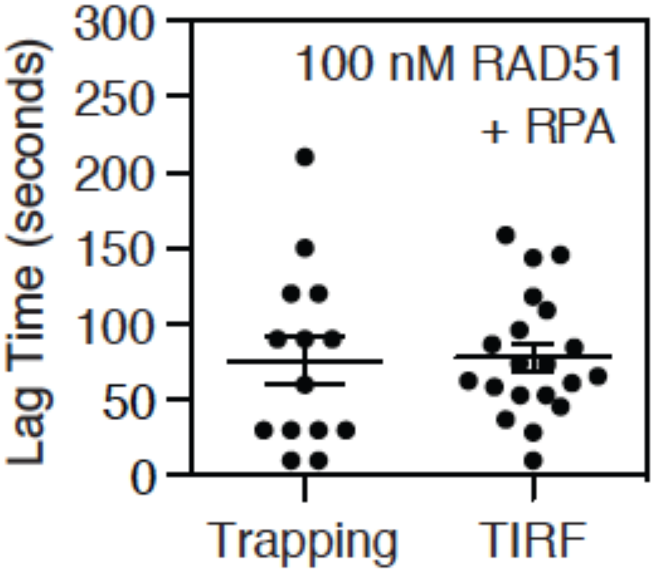

Supplementary Figure 2: Measurements of RAD51 nucleation using optical trapping and TIRF microscopy are indistinguishable. The lag time was plotted for molecules visualized using either optical trapping as described for Figure 1 (76±16 s, *N*=14) or TIRF as described in Figure 2 (78±9 s, *N*=14) with no observable difference in the distribution of lag times. Error is standard error of the mean.

Movie 1: Binding of BRCA2 on gapped λDNA. Movie of a single gapped λDNA molecule held between two optically trapped polystyrene beads in a 6-channel flow cell as described and shown in Supplementary Figure 1. The molecule was imaged in Channel 5 and iteratively dipped into Channel 6, containing BRCA2 (purple), between images in the montage. The BRCA2 appears to slide in the direction if flow (*left to right*) between 32 s and 64 s towards the junction between the dsDNA and RPA-coated ssDNA. At the end of the movie, the gapped λDNA was re-stained in Channel 3.

Movie 2: RAD51 binding to ssDNA in the absence of RPA using TIRF microscopy. A single gapped λDNA molecule was initially visualized using SYTOX Orange (*red*), which was subsequently dissociated upon the addition of binding buffer and fluorescent RAD51 (*green*) in the absence of RPA. Movie was collected as described in **Fig. 2A** and corresponds to the montage shown in **Fig. 2B**.

Movie 3: RAD51 binding to ssDNA in the presence of RPA using TIRF microscopy. A single gapped λDNA molecule was initially visualized using SYTOX Orange (*red*), which was subsequently dissociated upon the addition of binding buffer and fluorescent RAD51 (*green*) in the presence of RPA. Movie was collected as described in **Fig. 2A** and corresponds to the montage shown in **Fig. 2C**.
